# Insufficient evidence for an association between iatrogenic Alzheimer's disease and cadaveric pituitary‐derived growth hormone

**DOI:** 10.1002/alz.14127

**Published:** 2024-07-22

**Authors:** Avi Nath, David M. Holtzman, Bruce L. Miller, Lea T. Grinberg, Ellen Werber Leschek

**Affiliations:** ^1^ National Institute of Neurological Disorders and Stroke, National Institutes of Health Bethesda Maryland USA; ^2^ Department of Neurology Hope Center for Neurological Disorders Knight Alzheimer's Disease Research Center Washington University School of Medicine St. Louis Missouri USA; ^3^ UCSF Weill Institute for Neurosciences University of California San Francisco San Francisco California USA; ^4^ National Institute of Diabetes and Digestive and Kidney Diseases, National Institutes of Health Bethesda Maryland USA

**Keywords:** Alzheimer's disease, amyloid‐beta, cadaveric pituitary‐derived human growth hormone, cerebral amyloid angiopathy, Creutzfeldt‐Jakob disease, dementia, tau

## Abstract

A *Nature Medicine* paper published in January 2024 describes eight cases of iatrogenic Alzheimer's disease in individuals who received cadaveric pituitary‐derived human growth hormone. The paper's conclusions argue for the transmissibility of Alzheimer's disease, which, if true, would create a significant public health crisis. For example, neurosurgical practices would require substantial revision, and many individuals who have undergone neurosurgical procedures would now be at considerable risk of Alzheimer's disease. A detailed review of the presented cases reveals that they do not have Alzheimer's disease, and there are alternative explanations for the cognitive decline described. In people with progressive cognitive decline, the diagnosis of Alzheimer's disease requires a demonstration of amyloid and tau pathology or amyloid and tau biomarkers. Extensive tau pathology is not demonstrated, and some also lack amyloid beta pathology. The cases described in this paper do not meet the criteria for dementia due to Alzheimer's disease by clinical and pathological standards.

**Highlights:**

- Creutzfeldt‐Jakob disease has been transmitted by cadaveric growth hormone.
- There is no evidence for the transmission of Alzheimer's disease by cadaveric growth hormone.
- There is no evidence that Alzheimer's disease is transmissible.

1

On January 29, 2024, *Nature Medicine* published a manuscript entitled “Iatrogenic Alzheimer's Disease in Recipients of Cadaveric Pituitary‐Derived Growth Hormone.”^1^ In this paper, the authors describe eight cases of possible iatrogenic Alzheimer's disease (AD) in individuals who received cadaveric pituitary‐derived human growth hormone (c‐hGH) in the United Kingdom. This builds upon their previous work showing transmission of prions causing iatrogenic Creutzfeldt‐Jakob Disease (iCJD) and amyloid beta (Aβ) seeds that result in cerebral amyloid angiopathy (CAA).^2^

In this report, written by Banerjee et al., all patients received the Wilhelmi or Hartree‐modified Wilhelmi (HWP) batch of c‐hGH, which was previously associated with the transmission of iCJD.^1^ Also, this batch was found by the group to have amyloid seeds that could be transmitted to a transgenic mouse model, resulting in CAA‐like pathology.^2^ If Banerjee et al.’s current conclusions are correct, they would have profound implications for future work in this space. They suggest that other individuals who received the HWP batch of c‐hGH may be at risk of AD at an early age. Also, they imply that AD is transmissible, meaning that brain surgery and surgical practices (including tissue disposal) would need revision. Further, significant populations have already been exposed to this material and could be at risk, posing a potential public health crisis. Due to the implications of this study, it is critical that the cases presented in the manuscript be closely examined to understand whether they support a diagnosis of AD. A review of these cases suggests that most (if not all) do not have AD and there may be alternative explanations for the cognitive dysfunction in these cases. AD diagnosis in individuals with progressive cognitive decline requires demonstration of amyloid and tau pathology or amyloid and tau biomarkers.^3^, ^4^ Extensive tau pathology is lacking in most tests, and some also lack Aβ pathology. What follows is a brief discussion of each case as well as a summary table (Table 1).

**TABLE 1:** Summary of cases included in Banerjee et al.^1^

Case	Primary CNS lesion (treatment)	c‐hGH indication	Memory deficits	Brain imaging	CSF	Autopsy results
**1**	Craniopharyngioma (surgery, radiation)	GHD	No	N/A	N/A	Extensive Aβ pathology; tau pathology limited to insular cortex and absent in frontal and temporal regions
**2**	Craniopharyngioma (surgery)	GHD	Yes	N/A	Normal Aβ, elevated total, and phosphorylated tau	Amyloid deposits in neocortex; rare tau deposits
**3**	N/A	Silver‐Russell Syndrome	Yes	MRI: mild left temporal and left hippocampal atrophy Amyloid‐PET: widespread Aβ deposition in frontal, parietal, and temporal lobes	low amyloid, elevated total tau	N/A—still alive
**4**	Septo‐optic dysplasia	GHD	Yes	CT: generalized atrophy	N/A	N/A – still alive
**5**	Medulloblastoma (surgery, radiation, chemotherapy)	GHD	Yes	MRI: bi‐frontal atrophy	N/A	Not done
**6**	N/A	Idiopathic isolated GHD	No	MRI: normal	Low Aβ 42/40 ratio, elevated phosphorylated tau 181	N/A – still alive
**7**	N/A	Idiopathic isolated GHD	Yes	MRI: small solitary right temporal microhemorrhage Amyloid‐PET: normal	Normal Aβ 42/40 ratio and total tau	N/A – still alive
**8**	Craniopharyngioma (surgery, radiation)	GHD	Yes	CT: cerebellar atrophy	Normal Aβ, normal total, and phosphorylated tau	N/A – still alive

Abbreviations: Aβ, amyloid‐beta; c‐hGH, cadaveric pituitary‐derived human growth hormone; CT, computerized tomography; MRI, magnetic resonance imaging; GHD, growth hormone deficiency.

Case 1 had a craniopharyngioma treated with surgery and radiation at 3 and again at 5 years old due to recurrence. Additional recurrences occurred at ages 42 and 44. At 44 years old he experienced behavioral changes; memory and language were intact. The clinical picture was highly atypical for AD. Autopsy showed extensive Aβ pathology, but tau pathology was absent in the frontal and temporal regions and limited to the insular cortex.

Case 2 had a craniopharyngioma treated with surgery at 5 years old. The tumor recurred at 28 and was treated with surgery, radiation, and ventriculoperitoneal (VP) shunting. This patient also had a seizure disorder and focal weakness. At 55 years old there was an acute change in mental status with rapid cognitive decline over 15 months and subsequent death. The autopsy showed amyloid deposits confined to the neocortex (Thal phase 1, CERAD score 0) and only rare tau deposits, which did not meet the criteria for Braak stage 1.^5^ Tau pathology strongly correlates with clinical deficits in AD. This case and the lack of tau are highly atypical for dementia due to AD.

Case 3 had Russell‐Silver Syndrome and had meningococcal meningitis at 2 months old. Cognitive decline started at 38 years old and progressed over the subsequent 10 years. The amyloid positron emission tomography (PET) scan was positive, cerebrospinal fluid (CSF) Aβ peptide 42 was low, and CSF total tau was elevated. However, phospho‐tau (p‐tau) was not measured, and tau‐PET was not performed. This patient is alive (54 years old at time of publication). This patient may have AD, but a more comprehensive evaluation is needed to confirm the diagnosis.

Case 4 had congenital septo‐optic dysplasia. Cognitive decline started at 46 years old and progressed over the subsequent 4 years. A computerized tomography (CT) scan showed generalized atrophy, but no magnetic resonance imaging (MRI) or CSF evaluation was reported. This patient is still alive (49 years old at time of publication). Biomarkers are needed for an AD diagnosis.

Case 5 had a medulloblastoma at 3 years old treated with surgery, radiation, chemotherapy, and VP shunting. Intellectual disability was noted after treatment. At 10 years old she was involved in a serious motor vehicle accident, resulting in post‐traumatic epilepsy. She experienced progressive cognitive decline starting at 53 years old and died at 54. No autopsy was performed. MRI scan prior to death showed bifrontal atrophy. No CSF findings are reported. The etiology of this patient's dementia is unknown.

Case 6 had idiopathic isolated growth hormone deficiency and is enrolled in a longitudinal study for prion disease in c‐hGH recipients. This patient had normal neurocognitive testing and a normal brain MRI at 54 years of age. CSF Aβ peptide 42/40 ratio was low, and p‐tau 181 was elevated. This patient is still alive (54 years old at time of publication). It is possible that this patient has preclinical AD, which can last as long as 20 years.

Case 7 had idiopathic isolated growth hormone deficiency and seizure disorder and is enrolled in a longitudinal study for prion disease in c‐hGH recipients. He had an episode of meningococcal meningitis at 18 years old. He also has a mood disorder (ongoing severe anxiety and moderate depression). He developed subjective cognitive complaints at 52 years old. Neuropsychological testing showed normal cognitive performance, which remained stable during the 4 subsequent years. MRI showed a small, solitary microhemorrhage in the right temporal area. Amyloid PET was normal, and CSF was normal for amyloid peptides and tau. This patient is still alive (56 years old at time of publication). AD is highly unlikely as a cause of this patient's clinical issues.

Case 8 had a craniopharyngioma diagnosed at 6 years old treated with surgery and radiation. He experienced progressive cognitive decline starting at 48 years old. At 50, he was hospitalized with sepsis and adrenal crisis, and during the subsequent 3 years his cognitive issues continued to worsen and were accompanied by cerebellar and pyramidal tract signs. CT of the brain showed cerebellar atrophy, but CSF was normal for Aβ peptides, total tau, and p‐tau. This patient is still alive (55 years old at time of publication). It is highly unlikely that this patient has AD.

The presentations of these cases are highly variable and are not consistent clinically or pathologically with probable or definite AD. Only three patients underwent testing for genes associated with AD and other neurodegenerative diseases (those tested were negative). None had convincing extensive tau pathology; others had no evidence of amyloid pathology, and still others were not tested for either. Some of these patients are still alive, so additional investigations should be considered. For autopsied cases, tau pathology should be demonstrated in regions involved in cognitive decline to make a convincing argument that these are truly AD as opposed to findings attributable to the comorbidities present in these patients.

Public health implications would be profound if the patients reported by Banerjee et al. had a definitive AD diagnosis. Other individuals who received the HWP batch of c‐hGH could be at risk for AD at an early age. In addition, this would suggest that AD could be transmissible, which would have a significant impact on neurosurgical practices, including risks to patients, those performing the procedures, and individuals handling and being exposed to neurosurgical waste. Similarly, pathologists and technicians handling brain tissues from surgical procedures or at autopsy would also be at risk. Further, significant populations could have already been exposed to potentially infectious materials and thus would be at risk. All of these issues together would pose a public health crisis. Because of these serious potential consequences, it is critical that the cases described by Banerjee et al. be closely examined to determine whether they support the diagnosis of AD. As presented, most of these cases do not meet the criteria for diagnosis of AD according to well‐established clinical and pathological standards, which must be met to assign an AD diagnosis. Additionally, the highly variable clinical and pathological presentations and the likelihood of alternative diagnoses or explanations of the symptoms makes it difficult to link the presentations to the receipt of c‐hGH.

We conclude that, given the evidence presented, none of these cases can definitively be called symptomatic AD. Although the authors acknowledge the heterogeneity among the patients and discuss the possibility of atypical presentation, classifying these patients as having cognitive decline due to AD could be misleading. Significant public health implications are associated with the conclusions presented by Banerjee et al., which is why it is important to emphasize that these cases do not meet the criteria for dementia due to AD by clinical and pathological standards.

## CONFLICT OF INTEREST STATEMENT

None of the authors have conflicts or competing interests (financial or non‐financial) that affect the objectivity, integrity, and value of this publication. Author disclosures are available in the Supporting Information.

## Supporting information

Supporting Information
