# Supplementary material for: Insufficient evidence for an association between iatrogenic Alzheimer's disease and cadaveric pituitary‐derived growth hormone
Source: Alzheimers Dement. 2024 Jul 22;20(10):7399–402. doi: 10.1002/alz.14127 (PMC11485294; doi:10.1002/alz.14127)
Supplement: Supplementary file 1 — Supporting Information [file ALZ-20-7399-s001.pdf]

ICMJE DISCLOSURE FORM

**Date:**	6/13/2024
**Your Name:**	Ellen Werber Leschek
**Manuscript Title:**	Insufficient Evidence for an Association Between Iatrogenic Alzheimer's Disease and Cadaveric Pituitary-Derived Growth Hormone
**Manuscript Number (if known):**	AJD-D-24-00876

In the interest of transparency, we ask you to disclose all relationships/activities/interests listed below that are related to the content of your manuscript. "Related" means any relation with for-profit or not-for-profit third parties whose interests may be affected by the content of the manuscript. Disclosure represents a commitment to transparency and does not necessarily indicate a bias. If you are in doubt about whether to list a relationship/activity/interest, it is preferable that you do so.

The author's relationships/activities/interests should be defined broadly. For example, if your manuscript pertains to the epidemiology of hypertension, you should declare all relationships with manufacturers of antihypertensive medication, even if that medication is not mentioned in the manuscript.

In item #1 below, report all support for the work reported in this manuscript without time limit. For all other items, the time frame for disclosure is the past 36 months.

		**Name all entities with whom you have this relationship or indicate none (add rows as needed)**	**Specifications/Comments (e.g., if payments were made to you or to your institution)**
		Time frame: Since the initial planning of the work	
1	All support for the present manuscript (e.g., funding, provision of study materials, medical writing, article processing charges, etc.) **No time limit for this item.**	☒ None	
			Click the tab key to add additional rows.
		Time frame: past 36 months	
**2**	Grants or contracts from any entity (if not indicated in item #1 above).	☒ **None**	
**3**	Royalties or licenses	☒ **None**	

		Name all entities with whom you have this relationship or indicate none (add rows as needed)	Specifications/Comments (e.g., if payments were made to you or to your institution)
**4**	Consulting fees	☒ **None**	
**5**	Payment or honoraria for lectures, presentations, speakers bureaus, manuscript writing or educational events	☒ **None**	
**6**	Payment for expert testimony	☒ **None**	
**7**	Support for attending meetings and/or travel	☒ **None**	
**8**	Patents planned, issued or pending	☒ **None**	
**9**	Participation on a Data Safety Monitoring Board or Advisory Board	☒ **None**	
**10**	Leadership or fiduciary role in other board, society, committee or advocacy group, paid or unpaid	☒ **None**	

		Name all entities with whom you have this relationship or indicate none (add rows as needed)	Specifications/Comments (e.g., if payments were made to you or to your institution)
**11**	Stock or stock options	☒ **None**	
**12**	Receipt of equipment, materials, drugs, medical writing, gifts or other services	☒ **None**	
**13**	Other financial or non-financial interests	☒ **None**	

Please place an "X" next to the following statement to indicate your agreement:

☒ I certify that I have answered every question and have not altered the wording of any of the questions on this form.

ICMJE DISCLOSURE FORM

Date: 6/11/2021

Your Name: David M. Holtzman

Manuscript Title: Insufficient Evidence for an Association Between Iatrogenic Alzheimer's Disease and Cadaveric Pituitary-Derived Growth Hormone

Manuscript Number (if known): AJD-D-24-00876

In the interest of transparency, we ask you to disclose all relationships/activities/interests listed below that are related to the content of your manuscript. "Related" means any relation with for-profit or not-for-profit third parties whose interests may be affected by the content of the manuscript. Disclosure represents a commitment to transparency and does not necessarily indicate a bias. If you are in doubt about whether to list a relationship/activity/interest, it is preferable that you do so.

The author's relationships/activities/interests should be defined broadly. For example, if your manuscript pertains to the epidemiology of hypertension, you should declare all relationships with manufacturers of antihypertensive medication, even if that medication is not mentioned in the manuscript.

In item #1 below, report all support for the work reported in this manuscript without time limit. For all other items, the time frame for disclosure is the past 36 months.

		**Name all entities with whom you have this relationship or indicate none (add rows as needed)**	**Specifications/Comments (e.g., if payments were made to you or to your institution)**
**Time frame: Since the initial planning of the work**			
1	All support for the present manuscript (e.g., funding, provision of study materials, medical writing, article processing charges, etc.) **No time limit for this item.**	☒ None	
			Click the tab key to add additional rows.
**Time frame: past 36 months**			
**2**	Grants or contracts from any entity (if not indicated in item #1 above).	☐ **None**	
		NIH	GHR Foundation
		Eli Lilly	NextCure
		Cure Alzheimer's Fund	Novartis
		Ludwig Foundation	Ionis
		Centene	
		Rainwater Foundation	
**3**	Royalties or licenses	☐ **None**	
		License to NextCure for anti-APOE antibodies with royalties	

		Name all entities with whom you have this relationship or indicate none (add rows as needed)	Specifications/Comments (e.g., if payments were made to you or to your institution)
		License to C2N Diagnostics for methods to measure neurally derived molecules with royalities	
4	Consulting fees	☐ **None**	
		C2N Diagnostics	Asteroid
		Denali	
		Genentech	
		Cajal Neuroscience	
5	Payment or honoraria for lectures, presentations, speakers bureaus, manuscript writing or educational events	☐ **None**	
		Northwestern Univ - lecture	Arizona State Univ - lecture
		Cedars Sinai -lecture	
		Univ Iowa - lecture	
6	Payment for expert testimony	☒ **None**	
7	Support for attending meetings and/or travel	☒ **None**	
8	Patents planned, issued or pending	☐ **None**	
		Patent pending on Anti-MTBR 243 antibody	
9	Participation on a Data Safety Monitoring Board or Advisory Board	☒ **None**	
10	Leadership or fiduciary role in other board,	☒ **None**	

		Name all entities with whom you have this relationship or indicate none (add rows as needed)	Specifications/Comments (e.g., if payments were made to you or to your institution)
	society, committee or advocacy group, paid or unpaid		
**11**	Stock or stock options	☒ **None**	
**12**	Receipt of equipment, materials, drugs, medical writing, gifts or other services	☒ **None**	
**13**	Other financial or non-financial interests	☒ **None**	

Please place an "X" next to the following statement to indicate your agreement:

☒ I certify that I have answered every question and have not altered the wording of any of the questions on this form.

ICMJE DISCLOSURE FORM

**Date:**	6/13/2024
**Your Name:**	AVINDRA NATH
**Manuscript Title:**	Insufficient Evidence for an Association Between Iatrogenic Alzheimer's Disease and Cadaveric Pituitary-Derived Growth Hormone
**Manuscript Number (if known):**	AJD-D-24-00876

In the interest of transparency, we ask you to disclose all relationships/activities/interests listed below that are related to the content of your manuscript. "Related" means any relation with for-profit or not-for-profit third parties whose interests may be affected by the content of the manuscript. Disclosure represents a commitment to transparency and does not necessarily indicate a bias. If you are in doubt about whether to list a relationship/activity/interest, it is preferable that you do so.

The author's relationships/activities/interests should be defined broadly. For example, if your manuscript pertains to the epidemiology of hypertension, you should declare all relationships with manufacturers of antihypertensive medication, even if that medication is not mentioned in the manuscript.

In item #1 below, report all support for the work reported in this manuscript without time limit. For all other items, the time frame for disclosure is the past 36 months.

		**Name all entities with whom you have this relationship or indicate none (add rows as needed)**	**Specifications/Comments (e.g., if payments were made to you or to your institution)**
**Time frame: Since the initial planning of the work**			
1	All support for the present manuscript (e.g., funding, provision of study materials, medical writing, article processing charges, etc.) **No time limit for this item.**	☒ None	
			Click the tab key to add additional rows.
**Time frame: past 36 months**			
**2**	Grants or contracts from any entity (if not indicated in item #1 above).	☐ **None**	
		National Institutes of Health	
**3**	Royalties or licenses	☒ **None**	

		Name all entities with whom you have this relationship or indicate none (add rows as needed)	Specifications/Comments (e.g., if payments were made to you or to your institution)
**4**	Consulting fees	☒ **None**	
**5**	Payment or honoraria for lectures, presentations, speakers bureaus, manuscript writing or educational events	☐ **None**	
		Editorial Board, Update Neurology	Honoraria paid to me.
**6**	Payment for expert testimony	☒ **None**	
**7**	Support for attending meetings and/or travel	☒ **None**	
**8**	Patents planned, issued or pending	☐ **None**	
		Patent on antibody to human endogenous retrovirus envelope protein	Royalties paid to NINDS, NIH
**9**	Participation on a Data Safety Monitoring Board or Advisory Board	☒ **None**	
**10**	Leadership or fiduciary role in other board, society, committee or advocacy group, paid or unpaid	☐ **None**	
		Board member, American Neurological Association	unpaid
		Board member, International Society of Neurovirology	unpaid

		Name all entities with whom you have this relationship or indicate none (add rows as needed)	Specifications/Comments (e.g., if payments were made to you or to your institution)
**11**	Stock or stock options	☒ **None**	
**12**	Receipt of equipment, materials, drugs, medical writing, gifts or other services	☒ **None**	
**13**	Other financial or non-financial interests	☒ **None**	

Please place an "X" next to the following statement to indicate your agreement:

☒ I certify that I have answered every question and have not altered the wording of any of the questions on this form.

ICMJE DISCLOSURE FORM

Date: 6/17/2024

Your Name: Bruce L. Miller

Manuscript Title: Insufficient Evidence for an Association Between Iatrogenic Alzheimer's Disease and Cadaveric Pituitary-Derived Growth Hormone

Manuscript Number (if known): AJD-D-24-00876

In the interest of transparency, we ask you to disclose all relationships/activities/interests listed below that are related to the content of your manuscript. "Related" means any relation with for-profit or not-for-profit third parties whose interests may be affected by the content of the manuscript. Disclosure represents a commitment to transparency and does not necessarily indicate a bias. If you are in doubt about whether to list a relationship/activity/interest, it is preferable that you do so.

The author's relationships/activities/interests should be defined broadly. For example, if your manuscript pertains to the epidemiology of hypertension, you should declare all relationships with manufacturers of antihypertensive medication, even if that medication is not mentioned in the manuscript.

In item #1 below, report all support for the work reported in this manuscript without time limit. For all other items, the time frame for disclosure is the past 36 months.

		**Name all entities with whom you have this relationship or indicate none (add rows as needed)**	**Specifications/Comments (e.g., if payments were made to you or to your institution)**
**Time frame: Since the initial planning of the work**			
1	All support for the present manuscript (e.g., funding, provision of study materials, medical writing, article processing charges, etc.) **No time limit for this item.**	☒ None	
**Time frame: past 36 months**			
**2**	Grants or contracts from any entity (if not indicated in item #1 above).	☐ **None**	
		NIH/Univ. of Wisconsin, Madison	1R01AG070883
		NIH/NIA	R35AG072362
		Bluefield Project to Cure FTD, UCSF FTD Core	P0544014
		NIH/NIA	P30AG062422
		NIH/NIA	R01AG057234
		NIH/NIA	R01AG062562
		NIH/NIA	R01AG062588
		NIH CSR	R01AG052496

		Name all entities with whom you have this relationship or indicate none (add rows as needed)	Specifications/Comments (e.g., if payments were made to you or to your institution)
**3**	Royalties or licenses	☐ **None**	
		Cambridge University Press	Payment made to me
		Elsevier, Inc.	Payment made to me
		Guilford Publications, Inc.	Payment made to me
		Johns Hopkins Press	Payment made to me
		Oxford University Press	Payment made to me
		Taylor & Francis Group	Payment made to me
**4**	Consulting fees	☐ **None**	
		Massachusetts General Hospital Alzheimer's Disease Research Center (ADRC) Scientific Advisory Board (SAB)	Payments made to me in 2021, 2022, and 2023
		Stanford University ADRC SAB	Payments made to me in 2021, 2022, and 2023
		University of Washington ADRC SAB	Payments made to me in 2021, 2022, and 2023
		Genworth Medical Advisory Board	Payment made to me in March 2023
**5**	Payment or honoraria for lectures, presentations, speakers bureaus, manuscript writing or educational events	☐ **None**	
		Fromm Institute for Lifelong Learning	May 2023, payment made to me
		Global Summit on Neurodegenerative Diseases	Jun 2021, payment made to me
		Korean Dementia Society	Jul 2022, payment made to me
		Massachusetts General Hospital, dementia course	Payments made to me in 2022 and 2023
		National MS Society, Don Paty Lectureship	Jun 2021, payment made to me
		Ochsner Neuroscience Institute	Nov 2021, payment made to me
		Providence Saint Joseph Medical Center	Sep 2021, payment made to me
		Taipei Medical University, Dementia Center	Mar 2022, payment made to me
		UC Irvine Institute for Memory Impairments and Neurological Disorders (UCI MIND)	Mar 2022, payment made to me
		University of California, Los Angeles (UCLA) Grand Rounds	Apr 2022, payment made to me
		University of Texas, Center for Brain Health	Jan 2021, payment made to me
**6**	Payment for expert testimony	☒ **None**	
**7**	Support for attending meetings and/or travel	☐ **None**	
		The Association for Frontotemporal Degeneration (AFTD) Education Symposium, St. Louis, MO	May 2023, travel and lodging support
		Milken Institute FTD Scientific Retreat, Los Angeles, CA	Mar 2023, travel and lodging support
		California Institute of the Arts, Los Angeles, CA	Apr 2022, travel and lodging support
		UCLA	Apr 2022, travel and lodging support

		Name all entities with whom you have this relationship or indicate none (add rows as needed)	Specifications/Comments (e.g., if payments were made to you or to your institution)
**8**	Patents planned, issued or pending	☒ **None**	
**9**	Participation on a Data Safety Monitoring Board or Advisory Board	☐ **None**	
		Arizona Alzheimer's Consortium	External Advisor
		Association for Frontotemporal Degeneration	Scientific Advisor
		The Buck Institute for Research on Aging	Scientific Advisor
		Cure ALS	Scientific Advisor
		The John Douglas French Alzheimer's Foundation	Medical Advisor
		Fundación Centro de Investigación Enfermedades Neurológicas, Madrid, Spain	Scientific Advisor
		Genworth	Scientific Advisor
		Kissick Family Foundation	Scientific Advisor
		The Larry L. Hillblom Foundation	Scientific Advisor
		Massachusetts General Hospital ADRC	Scientific Advisor
		National Institute for Health Research Cambridge Biomedical Research Center and its subunit, the Biomedical Research Unit in Dementia	Scientific Advisor
		Stanford University ADRC	Scientific Advisor
		University of Southern California P01 Urban Air Pollution and Alzheimer's Disease: Risk, Heterogeneity, and Mechanisms	External Advisory Committee
		University of Washington ADRC	Scientific Advisor
**10**	Leadership or fiduciary role in other board, society, committee or advocacy group, paid or unpaid	☐ **None**	
		The Bluefield Project to Cure FTD	Director and Internal Advisor
		Global Brain Health Institute	Founding Director
		Institute for Neurodegenerative Diseases	Affiliated Faculty
		Tau Consortium of the Rainwater Charitable Fdtn.	Co-Director and Scientific Advisor
**11**	Stock or stock options	☒ **None**	
**12**	Receipt of equipment, materials, drugs, medical writing, gifts or other services	☒ **None**	

		Name all entities with whom you have this relationship or indicate none (add rows as needed)	Specifications/Comments (e.g., if payments were made to you or to your institution)
**13**	Other financial or non-financial interests	☒ **None**	

Please place an "X" next to the following statement to indicate your agreement:

☒ I certify that I have answered every question and have not altered the wording of any of the questions on this form.

ICMJE DISCLOSURE FORM

**Date:**	6/5/2024
**Your Name:**	Lea T. Grinberg
**Manuscript Title:**	**Insufficient Evidence for an Association Between Iatrogenic Alzheimer's Disease and Cadaveric Pituitary-Derived Growth Hormone**
**Manuscript Number (if known):**	ADJ-D-24-00876

In the interest of transparency, we ask you to disclose all relationships/activities/interests listed below that are related to the content of your manuscript. "Related" means any relation with for-profit or not-for-profit third parties whose interests may be affected by the content of the manuscript. Disclosure represents a commitment to transparency and does not necessarily indicate a bias. If you are in doubt about whether to list a relationship/activity/interest, it is preferable that you do so.

The author's relationships/activities/interests should be defined broadly. For example, if your manuscript pertains to the epidemiology of hypertension, you should declare all relationships with manufacturers of antihypertensive medication, even if that medication is not mentioned in the manuscript.

In item #1 below, report all support for the work reported in this manuscript without time limit. For all other items, the time frame for disclosure is the past 36 months.

		Name all entities with whom you have this relationship or indicate none (add rows as needed)	Specifications/Comments (e.g., if payments were made to you or to your institution)
**Time frame: Since the initial planning of the work**			
1	All support for the present manuscript (e.g., funding, provision of study materials, medical writing, article processing charges, etc.) **No time limit for this item.**	☐ None	
		NIH	institution
		Rainwahter Charitable Foundation	institution
		Weill Neurosciences Hub	C institution lick the tab key to add additional rows.
**Time frame: past 36 months**			
**2**	Grants or contracts from any entity (if not indicated in item #1 above).	☒ **None**	
**3**	Royalties or licenses	☒ **None**	

		Name all entities with whom you have this relationship or indicate none (add rows as needed)	Specifications/Comments (e.g., if payments were made to you or to your institution)
**4**	Consulting fees	☐ **None**	
		Guidepoint Inc	To me
**5**	Payment or honoraria for lectures, presentations, speakers bureaus, manuscript writing or educational events	☐ **None**	
		Medscape Inc	To me
**6**	Payment for expert testimony	☒ **None**	
**7**	Support for attending meetings and/or travel	☐ **None**	
		Alzheimer Association	
		Rainwater Charitable Foundation	
**8**	Patents planned, issued or pending	☒ **None**	
**9**	Participation on a Data Safety Monitoring Board or Advisory Board	☒ **None**	
**10**	Leadership or fiduciary role in other board, society, committee or advocacy group, paid or unpaid	☐ **None**	
		Global Brain Health institute	

		Name all entities with whom you have this relationship or indicate none (add rows as needed)	Specifications/Comments (e.g., if payments were made to you or to your institution)
**11**	Stock or stock options	☒ **None**	
**12**	Receipt of equipment, materials, drugs, medical writing, gifts or other services	☒ **None**	
**13**	Other financial or non-financial interests	☒ **None**	

Please place an "X" next to the following statement to indicate your agreement:

☒ I certify that I have answered every question and have not altered the wording of any of the questions on this form.
